# Impact of *Blastocystis* carriage and colonization intensity on gut microbiota composition in a non-westernized rural population from Colombia

**DOI:** 10.1371/journal.pntd.0013111

**Published:** 2025-05-12

**Authors:** Sergio Castañeda, Jeff Tomiak, Lee O’Brien Andersen, Claudia Patricia Acosta, Luis Reinel Vasquez-A, Christen Rune Stensvold, Juan David Ramírez

**Affiliations:** 1 Centro de Investigaciones en Microbiología y Biotecnología-UR (CIMBIUR), Facultad de Ciencias Naturales, Universidad del Rosario, Bogotá, Colombia; 2 Laboratory of Parasitology, Department of Bacteria, Parasites and Fungi, Statens Serum Institut, Copenhagen, Denmark; 3 University of Stavanger, Department of Chemistry, Bioscience, and Environmental Engineering, Stavanger, Norway; 4 Grupo de investigación en Genética Humana, Departamento de Ciencias Fisiológicas, Facultad de Ciencias de la Salud, Universidad del Cauca,; 5 Centro de Estudios en Microbiología y Parasitología, Facultad de Ciencias de la Salud, Universidad del Cauca, Popayán, Colombia; 6 Molecular Microbiology Laboratory, Department of Pathology, Molecular and Cell-Based Medicine, Icahn School of Medicine at Mount Sinai, New York city, New York, United States of America; Hokkaido University International Institute for Zoonosis Control, JAPAN

## Abstract

**Background:**

The role of *Blastocystis*, a common intestinal parasitic protist of humans and other animals, in human health and disease remains elusive. Recent studies suggest a connection between *Blastocystis* colonization, healthier lifestyles, and high-diversity gut microbiota. Nevertheless, studies concerning the relationship between *Blastocystis* colonization, its intensity, and gut microbiota composition -involving both bacterial and eukaryotic communities- remain limited.

**Methods:**

This study examines the impact of *Blastocystis* carriage and colonization intensity on gut microbiota composition in a rural community in Colombia. A total of 88 human samples were collected from the rural population of Las Guacas village, located in the Cauca department in southwest Colombia. We utilized 16S and 18S rDNA sequencing to analyze both bacterial and eukaryotic microbiota, comparing *Blastocystis*-positive and -negative individuals, as well as groups with varying *Blastocystis* colonization intensity (low, medium, high), to identify distinct microbiota profiles and differentially abundant taxa linked to each condition.

**Results:**

The analysis revealed significant differences between *Blastocystis*-positive and -negative individuals. In terms of bacterial composition and structure, *Blastocystis*-positive individuals exhibited distinct microbiota profiles, as shown by beta diversity analysis. Taxa associated with colonization included *Bacteroides*, *Prevotella*, *Oscillibacter*, *Faecalibacterium*, and *Alistipes*. Higher *Blastocystis* colonization intensity was associated with an increased abundance of taxa such as *Alistipes* and *Lachnospira*, while lower intensities correlated with beneficial bacteria such as *Akkermansia*. Regarding eukaryotic composition, beta diversity analysis revealed distinct profiles associated with *Blastocystis* colonization. Differentially abundant taxa, including *Entamoeba coli*, were more prevalent in *Blastocystis*-positive individuals, while *Blastocystis*-negative individuals exhibited a higher abundance of opportunistic fungi, such as *Candida albicans*. Machine learning models, including random forest classifiers, supported these findings, identifying *Faecalibacterium* and *Bacteroides* as predictors of *Blastocystis* colonization.

**Conclusions:**

These findings suggest that *Blastocystis* may modulate gut microbiota, contributing to microbial balance providing new insights into the ecological implications of *Blastocystis* in rural populations.

## Author summary

*Blastocystis* is a common intestinal protist found in humans and animals, but its role in health and disease remains unclear. Some studies suggest that *Blastocystis* colonization is associated with healthier lifestyles and diverse gut microbiota, yet little is known about how its colonization and intensity influence bacterial and eukaryotic gut communities. This study analyzed 88 samples from a rural community in southwest Colombia to explore these associations. Using DNA sequencing, we compared bacterial and eukaryotic microbiota between *Blastocystis*-positive and -negative individuals and across varying colonization intensities. *Blastocystis*-positive individuals exhibited distinct bacterial profiles, characterized by higher abundances of *Faecalibacterium*, *Alistipes*, and *Prevotella*. Higher colonization intensity was associated with *Lachnospira* and *Alistipes*, while lower intensities correlated with *Akkermansia*, a bacterium linked to gut health. Among eukaryotes, *Blastocystis*-positive individuals showed distinct profiles, including *Entamoeba coli*, while *Blastocystis*-negative individuals had higher levels of fungi, such as *Candida albicans*. These findings suggest that *Blastocystis* plays a role in shaping gut microbiota, potentially contributing to microbial balance. By uncovering these relationships, this study provides new insights into the ecological and health implications of *Blastocystis*, particularly in rural populations where its prevalence is significant.

## Introduction

*Blastocystis* is an ubiquitous intestinal protist, commonly detected in both humans and animals worldwide [1,2]. Despite its frequent occurrence in human stool samples, its role in human health has long been debated. Traditionally regarded a pathogen, emerging evidence suggests a more complex relationship, with studies indicating that *Blastocystis* may be a commensal organism or even provide benefits to gut health [3–8]. Recent research has identified *Blastocystis* as a biomarker of healthier lifestyles, positively influencing individualized host responses to diet and disease susceptibility [7]. However, it is important to note that the majority of studies on *Blastocystis* carriage focus on apparently healthy individuals, and research comparing its impact in diseased versus healthy populations is relatively limited [3–8]. *Blastocystis’* association with the gut microbiota is particularly interesting, as colonization appears to coincide with favorable shifts in microbial diversity and composition, shaped by a range of host factors, including immune status, lifestyle, and geographic location [5–9]. However, studies of *Blastocystis* in a microbiota context typically focus only on links to bacterial taxa, not other eukaryotic taxa [5,10–12]. Moreover, most studies on the impact of *Blastocystis* on microbiota ecology have been based on data from Westernized populations, which possess distinct socio-economic, demographic, dietary, and lifestyle conditions [3,8]. These factors shape a characteristic microbiota profile that differs from non-Westernized populations, particularly in rural areas [13,14]. Given these differences, rural populations may exhibit unique microbiota profiles that could potentially alter the interaction dynamics between *Blastocystis* and the host microbiota, highlighting the need for further investigation in these contexts.

Rural communities from non-westernized populations offer a unique and underexplored context for investigating interactions between *Blastocystis* and the gut microbiota. It has been observed that modern Westernized populations differ from traditional and agrarian populations in terms of gut microbiota composition [14]. Non-Western individuals tend to harbor a more diverse community of protists, which contributes to the overall diversity of the human gut. This distinction underscores the importance of studying non-Westernized populations to better understand the full spectrum of gut microbial diversity and the ecological roles of protists like *Blastocystis* [14]. Additionally, intestinal parasitic infections, including those caused by *Blastocystis*, are more prevalent in these settings, likely influenced by factors such as limited access to healthcare and clean water, and consumption of diets rich in unprocessed foods [12,15–17]. Despite this, there is a dearth of comprehensive studies examining *Blastocystis* and its microbiota interactions in rural populations, with most existing research focusing on specific subgroups, such as school-aged children, and primarily analyzing bacterial components using 16S rRNA sequencing [5,18,19]. The role of eukaryotic microorganisms, which may hold the key to obtaining a more detailed and comprehensive understanding of gut microbial interactions, has largely been overlooked in these studies.

Although data indicating that *Blastocystis* may alter the structure and composition of the gut microbiota exist [4–8], a more nuanced approach could be useful, considering not only its presence but also its intensity and the potential dose-dependent effects on microbial ecosystems. Studies on *Giardia* have shown that its presence induces changes in the host microbiota. Notably, shifts in microbial diversity and the abundance of specific taxa are linked to varying levels of *Giardia* intensity [20–22]. For example, Mejia (2020) found that children with *Giardia* DNA concentrations greater than 1 fg/µl exhibited significantly lower microbial diversity and a higher abundance of *Prevotella* compared with non-infected children [20]. This suggests that parasite infection intensity (referred to as ‘intensity’ in the following) may be a critical factor influencing microbiota structure and composition.

This study aimed to address a key gap by investigating the relationship between *Blastocystis* colonization, its intensity, and gut microbiota composition—including both bacterial and eukaryotic communities—in individuals from a non-Westernized rural community in Colombia, South America. Specifically, we seek to determine whether *Blastocystis* colonization is associated with shifts in microbial diversity and the presence of key taxa that could impact host health.

## Methods

### Ethics statement

Informed written consent was obtained from every participant or their respective parent or caregiver. Formal written consent was obtained from the parent/guardian. The study design and research protocols received approval from the Bioethics Committee of Universidad del Cauca, Colombia (Project ID 5735).

### Study population

The study was conducted in Las Guacas, a rural village in the municipality of Popayán, southwest Colombia (S1 Fig) [17]. This community was selected due to its high prevalence of intestinal parasites and its socio-environmental conditions, characterized by inadequate sanitation, poor hygiene, and limited access to potable water. Participants were approached through local community leaders and health outreach programs. Informed written consent was obtained from every participant or their respective parent or caregiver. Formal written consent was obtained from the parent/guardian. The study design and research protocols were approved by the Bioethics Committee of Universidad del Cauca, Colombia [17]. A non-probabilistic convenience sampling approach was employed, and 88 stool samples were collected from healthy children and caregivers who agreed to participate in the study and provided a fecal sample.

### Stool sample collection

Each stool sample was obtained using sterile gloves, deposited in a sterile container, and transported under refrigeration at temperatures between 2–8°C. Upon arrival at the laboratory, it was then stored in ultra-freezers at -80°C for subsequent analysis. DNA extraction from stool samples was carried out using the Norgen fecal DNA extraction kit (Norgen Biotek Corp., Thorold, Ontario, Canada), which included an initial disruption step.

### Microbiome sequencing and analysis

#### Primer design.

The 16S rDNA gene was targeted for amplification, using a modified version of the published universal prokaryotic primers 341F/806R, targeting the V3-V4 hyper-variable regions [23,24]. The forward primer had three additional nucleotides attached in the 5’ end (ACTCCTAYGGGRBGCASCAG, 341F3) and the reverse primer had five additional nucleotides attached in the 5’ end (AGCGTGGACTACNNGGGTATCTAAT, 806R5). These changes were made to comply with the annealing temperature parameters of our standard PCR program.

The 18S rDNA gene was selected as target gene to ensure that a broad spectrum of eukaryotic species (parasites and fungi) would be amplified with as few primer sets as possible when assuming that the 18S rDNA sequence would be the most inter-species conserved gene [24]. 18S rDNA sequences were aligned for each species or genus and a consensus sequence was generated. The consensus sequences for each species were used for phylogenetic analysis in order to group the parasites according to their 18S rDNA sequence similarity rather than taxonomy. The consensus sequence from all species within each group was then aligned and primers were designed, to amplify all species within each group. Additionally, a consensus sequence from human 18S rDNA was aligned with each group, in order to design primers that would preferentially amplify non-human 18S rDNA. Three different primer sets were chosen, G3F1/G3R1 (GCCAGCAGCCGCGGTAATTC/ACATTCTTGGCAAATGCTTTCGCAG), G4F3/G4R3 (CAGCCGCGGTAATTCCAGCTC/GGTGGTGCCCTTCCGTCAAT) and G6F1/G6R1 (TGGAGGGCAAGTCTGGTGCC/ACGGTATCTGATCGTCTTCGATCCC). G3 and G6 primers are targeting the hyper-variable regions V3-V4 and G4 is targeting the hypervariable region V3-V5 of the 18S rDNA gene. Each primer set was aligned using BLAST to the NCBI database, using NCBI’s Primer-Blast, with standard settings (excluding predicted Refseq transcripts and uncultured/ environmental samples).

#### Library preparation and sequencing.

Purified genomic DNA from each sample was initially amplified using the 16S and three different 18S primers. For each primer, the rDNA was amplified using a short PCR setup; Initial denaturation at 95°C for 3 min, 20 cycles of 95°C (16S: for 30 sec; 18S: for 1 min), 60°C for 1 min, and 72°C for 30 sec, and a final elongation at 72°C (16S: for 7 min; 18S: for 4 min). PCR was performed in a 25µl volume, using the PrimeSTAR HS DNA Polymerase premix (Takara Bio, Shiga, Japan) with 0.4µM of each primer and 2µl template. This PCR is referred to as PCR1 or amplification PCR. The products from PCR1 were prepared for sequencing by a second PCR (PCR2 or adaptor PCR) using the same PCR program. PCR2 attaches adaptor A, an index i5, and a forward sequencing primer site (FSP) in the 5’ end of the amplicons and an adaptor B, an index i7, and a reverse sequencing primer site (RSP) to the 3’ end of the amplicons. Hence, four different PCR amplicon products were generated for each sample. DNA was quantified using the Quant-IT dsDNA High Sensitive Assay Kit (Thermo Fisher Scientific), and PCR2 products were pooled in equimolar amounts between samples into the pooled amplicon library (PAL). Undesirable DNA amplicons were removed from the PAL by Agencourt AMPure XP bead purification in a two-step process. Firstly, DNA fragments below 300nt were removed by a 10µL PAL to 24µL AMPure beads ratio, following the manufactures protocol and eluted in 40µL TE buffer. Secondly, large DNA fragments above 1kbp were removed by 10µL AM1–16µL AMPure beads ratio, as previously described. The resulting AMPure beads purified PAL was denoted bPAL. The bPAL was diluted to its final concentration of 7.5pM DNA with a 0.001 N NaOH concentration, used for sequencing on the Illumina MiSeq desktop sequencer (Illumina Inc., San Diego, CA 29122, USA). The library was sequenced with the 500-cycle MiSeq Reagent Kit V2 in a 2x250nt setup (Illumina Inc., San Diego, CA 29122, USA).

#### Data analysis.

The data analysis was performed using BION, a semi-commercial software package that utilizes a k-mer-based approach for species-level annotation [24]. BION can handle non-overlapping paired end reads and custom reference databases. It accepts raw sequences and includes several processing steps: de-multiplexing, primer extraction, sampling, sequence and quality-based trimming and filtering, de-replication, clustering, chimera checking, reference data similarity assessment, and taxonomic mapping and formatting.

Prokaryotic sequences were mapped against the RDP reference database (v11.04), while eukaryotic sequences were mapped using the SILVA reference database (v128). For eukaryotes, an in-house improved taxonomy definition was applied, aligning with prokaryotic taxonomy levels (phylum, class, order, family, genus, species, and sequence levels) [8,24–26]. Taxonomy profiling was conducted with an 85% k-mer similarity cut-off at the species level, corresponding to approximately 98% sequence similarity. At the genus level, the cut-off was set at 60% k-mer similarity, equivalent to around 95% sequence similarity. Query sequences with k-mer similarity below 60% were excluded from further analysis. Mapped query sequences were clustered at 98% k-mer similarity, providing a resolution higher than the basecalling error rate.

Microbial diversity analysis was performed using the R Phyloseq package [27]. This package was employed for calculating diversity and abundance indices and generating the corresponding graphs. Linear discriminant analysis (LDA) effect size (LEfSe) analysis and DESeq2 applying shrinkage estimation and Benjamini-Hochberg correction to adjust for multiple comparisons, were employed to identify taxa with differential abundance [28]. The threshold of the logarithmic LDA score was 4.0 with a maximum kw value of 0.01 for LEfSe analysis. For DEseq2, a pCutoff of 0.05 and an FCcutoff of 1.0 were used.

### *Blastocystis* positive and negative classification and intensity levels

To assess *Blastocystis* colonization status (positive or negative) and categorize colonization intensity, we analyzed sequencing data generated using Illumina technology, which targeted the G3, G4, and G6 regions of the 18S rDNA gene, following previously established protocols [8,26,29]. Colonization status was determined based on read counts obtained from the target regions. We first evaluated the distribution of read counts across all samples to establish a threshold for distinguishing *Blastocystis* presence from absence. An empirical threshold of 100 reads was established to classify samples as either *Blastocystis*-negative (≤100 reads) or *Blastocystis*-positive (>100 reads). This threshold was determined through a detailed analysis of read count distributions and alignment accuracy, which facilitated the precise assignment of subtypes (STs). The selection of this cutoff also aligns with recommendations from the relevant literature [30]. This cutoff was chosen to minimize false positives (e.g., due to technical contamination) while maintaining sensitivity to detect true colonization. For *Blastocystis*-positive samples, we categorized the intensity into three levels (low, medium, or high) using a modified quartile-based approach. Specifically, individuals in the 0–25th percentile were classified into the “Low” intensity group, those in the 25th-75th percentile were assigned to the “Middle” intensity group, and individuals with read counts exceeding the 75th percentile were placed in the “High” intensity group. This classification was applied to ensure a statistically meaningful distribution across groups, allowing for comparisons that account for variability in read counts. The corresponding read count ranges were defined as follows: Negative (0–100 reads), Low (110–694 reads), Middle (694–6118 reads), and High (more than 6118 reads). To reduce skewness and improve the normality of the read count data, a natural logarithmic transformation (ln) was applied to the positive read counts. This transformation helped to compress the range of values and diminish the influence of extreme outliers, making the data more suitable for parametric statistical analyses. The log-transformed intensity levels were as follows: Negative (0-4.61), Low (4.62-6.52), Middle (6.53-8.76), and High (8.77 and above). As a sensitivity check, we tested alternative cutoffs (e.g., tertiles and different percentile thresholds) and found that the overall trends in microbiota composition remained consistent, supporting the robustness of our classification approach. By employing this classification framework and normalization procedure, we provided a robust statistical basis for analyzing *Blastocystis* colonization and intensity across the study population.

### Statistical analysis

The quantitative variables were summarized in terms of medians and interquartile range, and the qualitative variables were summarized in frequencies and proportions. The number of reads in each sample was normalized using the average sequence depth. As indicators for the comparisons between groups, relative abundance, and alpha diversity metrics, observed richness, Shannon, and Fisher index, were used [31]. The alpha diversity indices were compared between groups using the non-parametric Mann-Whitney-Wilcoxon and Kruskal Wallis test. To explore the differences in the general composition of the microbial community between groups (beta diversity), the Bray-Curtis taxonomic distances were calculated. Using the *Phyloseq* package, a principal coordinates analysis diagram was produced, in which the Bray-Curtis dictations were used between the samples to visualize the behavior of the groups. Permutational analysis of variance (PERMANOVA) test for multivariate effect was done using the adonis function from the vegan package [32], stratified by groups. Statistical analyses were carried out using the R software [33]. For all continuous values, normality hypotheses were evaluated using the Kolmogorov-Smirnov test. All tests of significance were two-tailed, and *P*-values < 0.05 were considered statistically significant.

### Machine learning model

We employed a machine learning-based classification approach to predict the variable of interest from microbiota composition. Specifically, a random forest classifier, a supervised method designed to optimize classification accuracy, was utilized [34,35]. The dataset was analyzed in R using ggplot2, phyloseq, randomForest, and pROC packages. Samples were filtered to include only those with *Blastocystis* group status (Positive or Negative), and Operational Taxonomic Units (OTUs) from the Blastocystidae family were removed. In this context, OTUs are used as a general term to refer to the taxonomic features identified through amplicon sequencing. However, since the DADA2 pipeline was used in this study, the actual units analyzed are Amplicon Sequence Variants (ASVs), which represent exact sequence variants rather than clustered sequences based on similarity thresholds. For consistency and simplicity, we refer to these features as OTUs throughout the manuscript, acknowledging that they correspond to ASVs.

Rare OTUs were pruned based on a relative abundance threshold of 0.01%. The random forest model, consisting of 100 trees, was trained to classify Blastocystis status, and the 20 most important OTUs were identified using the Mean Decrease in Gini Index. Model performance was evaluated through a confusion matrix, precision, recall, F1 score, specificity, and ROC curve with AUC values. Visualization of OTUs was performed using ggplot2.

## Results

### General microbiota profile

**Fig 1:**
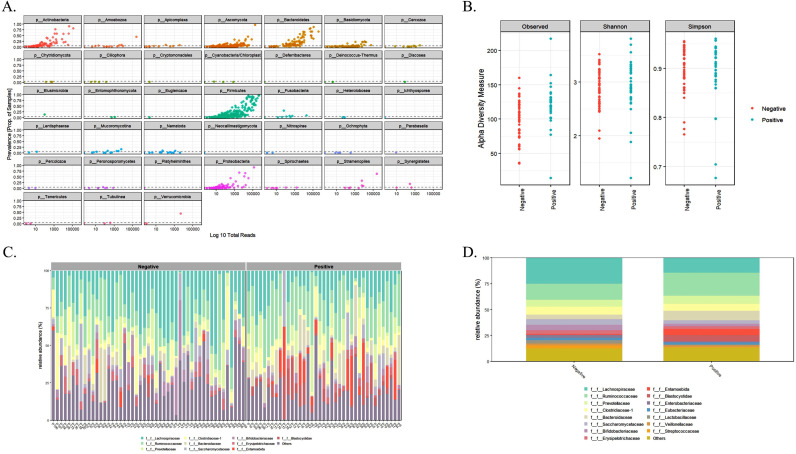
(A) Proportion of samples by total reads showing abundance by phylum. (B) Diversity metrics—richness, Shannon, and Simpson indices—of *Blastocystis*-positive and -negative groups. (C) Bar chart displaying the relative abundance of the top taxa at the family level for individual samples in *Blastocystis*-positive and -negative groups. (D) Bar chart showing the relative abundance of the top taxa at the family level aggregated by group. This figure was created using RStudio software.

Out of the 88 total samples analyzed (100%), *Blastocystis* was identified in 39 samples (44.3%), while 49 samples (55.7%) were negative. The composition and diversity of intestinal microbiota among participants with and without *Blastocystis* were evaluated using together 16S and 18S rDNA (Fig 1). The general profile showed that Firmicutes was the dominant Phyla, followed by Bacteroidetes, Proteobacteria, Actinobacteria, Ascomycota, and Amoebozoa (Fig 1A). Within these phyla, the most abundant bacterial family taxa were Lachnospiraceae and Rimunococcaceae, and for eukaryotes, Saccharomycetaceae and Entamoebidae (Fig 1C, 1D).

### Bacterial microbiota composition

*Alpha and beta diversity analyses revealed potential differences in bacterial composition between* Blastocystis*-positive and* Blastocystis*-negative individuals.*

**Fig 2:**
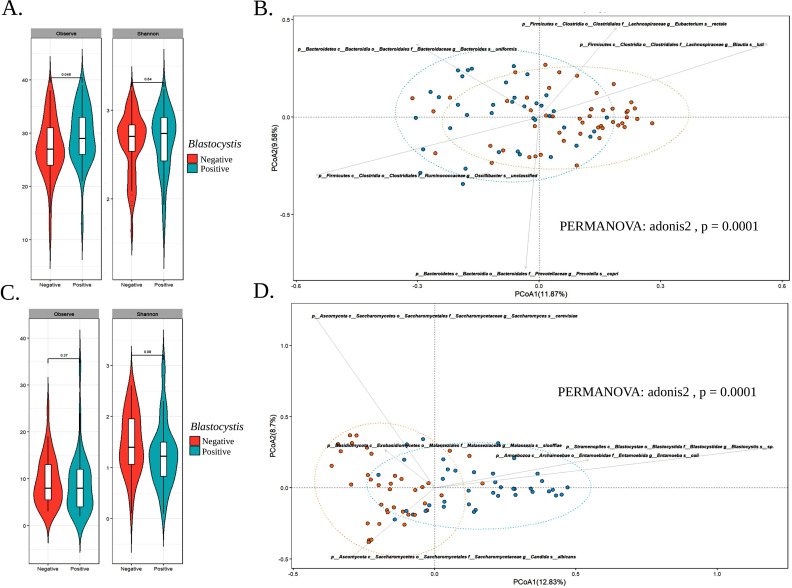
(A) Observed richness and Shannon indices for *Blastocystis*-positive and *Blastocystis*-negative groups based on bacterial profiles. Statistical significance was determined using the Kruskal-Wallis test, followed by a post-hoc Mann-Whitney-Wilcoxon test for multiple comparisons. Data are presented as interquartile ranges (IQR; boxes), with medians (lines within the boxes) and whiskers indicating the range within 1.5 times the IQR. (B) Principal coordinates analysis (PCoA) of Bray-Curtis distances for bacterial communities, comparing *Blastocystis*-positive and *Blastocystis*-negative groups. Permutational analysis of variance (PERMANOVA) was conducted using the adonis function from the vegan package. (C) Observed richness and Shannon indices for *Blastocystis*-positive and *Blastocystis*-negative groups based on eukaryotic profiles, analyzed as in (A). (D) Principal coordinates analysis (PCoA) of Bray-Curtis distances for eukaryotic communities, analyzed as in (B). *** p-values <0.001. This figure was created using RStudio software.

To evaluate differences in bacterial alpha diversity, we calculated observed richness and Shannon indices (Fig 2A). While the overall composition of the intestinal bacterial community did not differ significantly between groups, there was a trend toward higher observed richness in the *Blastocystis*-positive group. Significant differences in diversity metrics were identified across varying levels of *Blastocystis* intensity. Specifically, the group with medium *Blastocystis* intensity demonstrated a higher observed richness compared to the *Blastocystis*-negative group (S2A Fig). Nonetheless, beta diversity analysis revealed significant distinctions (PERMANOVA: adonis2, *P* = 0.0001) between *Blastocystis*-positive and -negative individuals, indicating the existence of two distinct microbiota profiles based on *Blastocystis* presence. Notably, in *Blastocystis*-positive individuals, taxa such as *Bacteroides uniformis*, *Oscillibacter* sp., and *Prevotella copri* were more abundant, while *Eubacterium rectale* and *Blautia luti* were more prevalent in *Blastocystis*-negative individuals (Fig 2B).

#### Microbiota bacterial composition: relative abundances and differential taxa.

To understand the global composition of the microbiota according to *Blastocystis* status, we analyzed relative abundances and differential taxa. Both *Blastocystis*-positive and -negative groups were predominantly characterized by the families Lachnospiraceae, Ruminococcaceae, Clostridiaceae, Prevotellaceae, and Bacteroidaceae. However, the *Blastocystis*-positive group exhibited a subtle shift, with a reduction in the relative abundance of Lachnospiraceae and an increase in Ruminococcaceae and Bacteroidaceae, suggesting specific microbial alterations associated with *Blastocystis* colonization (S3 Fig). When examining relative abundances based on *Blastocystis* intensity levels, these patterns became more pronounced in the high-intensity group, where the abundance of Ruminococcaceae and Bacteroidaceae was notably higher compared to other groups.

**Fig 3:**
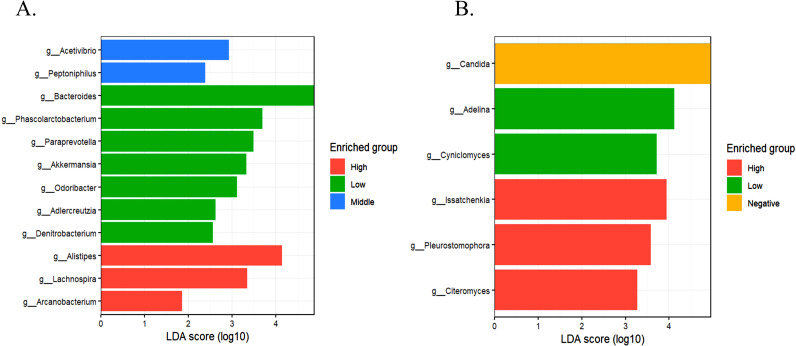
Linear discriminant analysis effect size (LEfSe) identifying taxa enriched in intensity groups of *Blastocystis*-positive samples, corresponding to (A) 16S rRNA data and (B) 18S rRNA data. The threshold for the logarithmic LDA score was 2.0, with a maximum Kruskal-Wallis (KW)/Wilcoxon Test value of 0.01. This figure was created using RStudio software.

We employed Linear Discriminant Analysis Effect Size (LEfSe) and DESeq2 to analyze the taxa differentially abundant among the comparison groups. Our comparison between *Blastocystis*-positive and -negative groups revealed that the *Blastocystis*-positive group exhibited higher abundances of genera such as *Bacteroides*, *Faecalibacterium*, and *Alistipes*. In contrast, the *Blastocystis*-negative group showed a greater prominence of genera like *Blautia*, *Staphylococcus*, *Enterococcus*, and *Klebsiella* (S3A Fig). Further analysis of *Blastocystis* intensity groups (high, medium, and low) identified distinct taxa associated with each intensity level. High intensity levels were linked to genera such as *Alistipes*, *Lachnospira*, and *Arcanobacterium*; medium intensity levels were associated with *Acetivibrio* and *Peptoniphilus*; and low intensity levels correlated with *Bacteroides*, *Phascolarctobacterium*, *Paraprevotella*, and *Akkermansia* (Fig 3A). These findings suggest that specific alterations in the microbiota may be linked not only to the presence of *Blastocystis* but also to the intensity of *Blastocystis*. This implies that variations in *Blastocystis* intensity could have a relevant impact on the dynamics underlying changes in the composition and structure of the host microbiota.

### Eukaryotic microbiota composition

*Beta diversity analysis of the eukaryotic microbiota also showed differences between* Blastocystis*-positive and -negative groups.*

Although alpha diversity indices did not show significant differences between *Blastocystis*-positive and -negative groups, our analysis according to *Blastocystis* intensity revealed that higher intensity was associated with reduced eukaryotic diversity compared with the *Blastocystis*-negative group (Mann-Whitney-Wilcoxon test, P < 0.05) (Fig 2C). This suggests that the presence of *Blastocystis* may inhibit the abundance of other eukaryotic organisms, particularly fungi, in the host microbiota or that at least it could have a better opportunity to expand. Beta diversity analysis further confirmed significant differences in eukaryotic microbiota composition between *Blastocystis*-positive and -negative individuals (PERMANOVA: adonis2, P = 0.0001), indicating distinct eukaryotic profiles associated with *Blastocystis* presence. Specifically, *Entamoeba coli* was commonly found in *Blastocystis*-positive individuals, whereas the fungi *Saccharomyces cerevisiae* and *Candida albicans* were more characteristic of the *Blastocystis*-negative group (Fig 2D).

### Eukaryotic microbiota composition: relative abundances and differential taxa

In terms of eukaryotic microbiota, both groups were predominantly characterized by the families Saccharomycetaceae, Blastocystidae, Entamoebidae, Malasseziaceae, and Trichomycetes. In the *Blastocystis*-positive group, we observed a notable reduction in the relative abundance of Saccharomycetaceae and an increase in Entamoebidae (Fig 1C, 1D). Regarding *Blastocystis* intensity groups, we observed that higher *Blastocystis* intensity was associated with a reduction in the relative abundance of other eukaryotes, particularly fungi from the families Saccharomycetaceae and Malasseziaceae. Notably, the family Entamoebidae consistently maintained a higher relative abundance across all intensity levels in *Blastocystis*-positive individuals.

To identify differentially abundant eukaryotic taxa for each group, we employed LEfSe and DESeq2. By comparing *Blastocystis*-positive and -negative groups, we found that the *Blastocystis*-positive group exhibited a differential abundance of genera such as *Barnettozyma*, *Entamoeba*, and *Endolimax* (S3B Fig). In contrast, the *Blastocystis*-negative group was characterized by higher abundances of *Candida* and *Malassezia* (S3B Fig). Additionally, analysis of *Blastocystis* intensity groups revealed that high intensity levels were associated with genera such as *Issatchenkia*, *Pleurostomophora*, and *Citeromyces*, whereas low intensity levels were related with genera like *Adelina* and *Cyniclomyces* (Fig 3B).

### Microbiota composition as a predictor of *Blastocystis* presence

**Fig 4:**
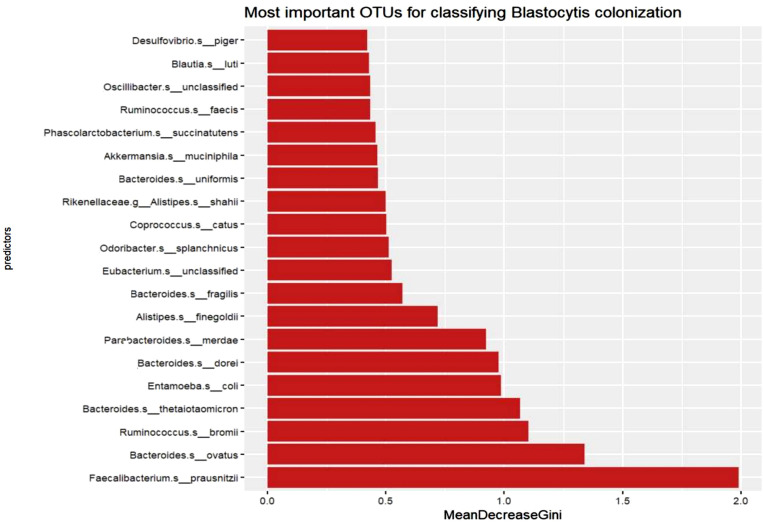
A random forest model was trained to classify *Blastocystis* colonization status. The figure highlights the 20 most important taxa identified based on the Mean Decrease in Gini Index, reflecting their contribution to the model’s predictive accuracy. This figure was created using RStudio software.

The random forest classifier was trained to predict *Blastocystis* colonization status based on microbiota composition (Fig 4). The model achieved an out-of-bag (OOB) error rate of 23.86%, indicating the proportion of samples misclassified during the internal validation process. The confusion matrix revealed that the model correctly classified 41 of 49 negative samples (class error = 16.3%) and 26 of 39 positive samples (class error = 33.3%). When evaluating the model on the test data, it achieved a perfect accuracy of 1.0, with a 95% confidence interval of (0.9589, 1). Additionally, the P-value for accuracy being greater than the No Information Rate (NIR) was highly significant (P < 2.2e-16), indicating that the model’s performance was far superior to random chance. These results suggest that the random forest model was highly effective in classifying *Blastocystis* colonization status based on microbiota composition, although there was some degree of misclassification in the training process, particularly among positive samples.

The random forest model identified the top 20 taxa that were most important for classifying *Blastocystis* colonization based on the Mean Decrease in Gini Index, which reflects each taxa contribution to the accuracy of the model. A bar plot of the Mean Decrease in Gini values revealed that taxa predictors varied significantly in their importance (Fig 4). The taxa with the highest Mean Decrease in Gini were the most influential in differentiating between positive and negative *Blastocystis* colonization. This visualization highlights the specific taxa that play a key role in driving the model’s predictive performance, indicating potential microbial markers associated with *Blastocystis* status.

## Discussion

Our findings suggest that *Blastocystis* colonization is potentially related with significant features in both the composition and structure of the gut microbiota, which may correlate with improvements in host health observed in non-westernized rural populations in previous studies (Fig 1) [7]. Piperni et al. (2024) reported that the changes in the gut microbiome related to *Blastocystis* vary across different geographical regions and lifestyles, highlighting a strong connection with healthier, plant-based diets common in these populations [7]. Additionally, their study indicated that *Blastocystis* was associated with improved cardiometabolic profiles, and its presence increased following a diet-improvement intervention program [7]. These findings could indicate that *Blastocystis* may have a potential impact on the host—not only in terms of microbiome composition and structure but also regarding metabolic and immunological processes that contribute to improved health conditions [7]. These results are particularly relevant for non-westernized rural populations, such as the Colombian cohort used in the present study, where environmental exposures, dietary practices, and lifestyle factors differ significantly from those in urban settings [8,13,14]. Such differences may potentially influence microbiota diversity and parasite prevalence. In this context, we found that the presence of *Blastocystis* was related to increased bacterial diversity, which aligns with previous research [7,8]. This suggests that *Blastocystis* may play a role in promoting a more diverse and stable microbial community. Greater microbial richness is often associated with improved health outcomes, as a more diverse microbiota provides functional redundancy, enhancing the gut’s resilience to disruptions such as infections or dietary changes [36–38].

Beta diversity analysis revealed that the bacterial microbiota exhibited a distinct profile concerning *Blastocystis* presence, suggesting that *Blastocystis* may modulate both the structure and composition of the gut microbiota (Fig 2A, 2B). The analysis also showed that among *Blastocystis*-positive individuals, certain bacterial taxa, such as *Bacteroides uniformis*, *Oscillibacter* sp., and *Prevotella copri*, were more prevalent, consistent with findings from previous studies [3,5,11,39]. *Bacteroides uniformis* is well-known for its role in carbohydrate metabolism and energy extraction from plant-based diets, contributing to nutrient absorption and gut barrier integrity [40–42]. In this context, *B. uniformis* has been shown to improve whole-body glucose disposal, increase butyrate production, and restore the balance of intraepithelial lymphocytes and type-3 innate lymphoid cells in the intestinal epithelium. These functions enhance both the metabolic and immunological properties of the gut barrier, which is particularly beneficial in non-westernized rural populations with higher fiber intake [40]. *Oscillibacter* has been associated with anti-inflammatory effects and may help protect against gut inflammation and promote overall well-being [8,43]. In addition to these properties*, Oscillibacter* has demonstrated potential benefits for lipid homeostasis and cardiovascular function. By contributing to the regulation of lipid metabolism, it may play a key role in maintaining metabolic balance, further supporting its protective effects on the host’s overall condition [8,43]. *Prevotella copri*, although associated with inflammation in certain contexts, is a key indicator of diets rich in fiber and plant-based foods [14]. *Prevotella copri* is also recognized as an important SCFA-producing bacterium, which plays a crucial role in maintaining proper metabolic functions in the gut and supporting energy processes in enterocytes [44,45]. The increased abundance of SCFA-producing taxa in *Blastocystis*-positive individuals suggests that *Blastocystis* may enhance these beneficial microbial functions, contributing to gut health.

These findings underscore the complex relationship between *Blastocystis* colonization, diet, and gut bacterial microbiota, suggesting how the presence of *Blastocystis* may contribute to microbiota diversity by promoting taxa linked to improved metabolic and immune functions. This interaction could have positive health implications, particularly for individuals consuming fiber-rich diets, as the promoted taxa may enhance nutrient absorption, immune responses, and gut homeostasis. These effects are especially relevant in non-westernized rural populations, where diet and lifestyle play a significant role in shaping microbiota composition [7,8,40,42,45–47]. *Blastocystis* could serve as a biomarker of a microbiome environment favoring resilience and diversity rather than actively inducing these effects. Additionally, although dietary profiles have been proposed as key modulators of *Blastocystis* colonization, our study does not directly assess dietary intake, and further research is needed to elucidate these associations. Furthermore, *Blastocystis* intensity levels appeared to be related to specific microbial taxa, suggesting a potential dose-dependent relationship between *Blastocystis* intensity and its impact on the microbiota (Fig 3A). In examining *Blastocystis* intensity groups (high, medium, and low), higher *Blastocystis* intensity was related to taxa such as *Alistipes* and *Lachnospira*, both known for their anti-inflammatory properties and role in maintaining gut homeostasis [48–50]. *Alistipes*, in particular, has been linked to a reduced risk of inflammation and metabolic disorders, suggesting that higher *Blastocystis* levels, along with other host conditions, may confer protective effects against inflammatory processes [49]. In contrast, lower *Blastocystis* intensity was associated with taxa such as *Akkermansia*, a bacterium recognized for maintaining the gut mucus layer and improving metabolic health. These findings suggest that the interaction between *Blastocystis* and the microbiota is complex and potentially modulated by the protist’s abundance in the gut [51]. To accurately assess these relationships, it is essential to develop study designs and protocols based on longitudinal cohorts. Such approaches would allow for a more dynamic evaluation of *Blastocystis* colonization and intensity over time and its potential impact on gut microbiota composition. Future studies should explore how dietary patterns influence *Blastocystis* colonization and intensity, particularly by comparing populations with predominantly vegetarian diets to those consuming meat-rich diets. Such comparisons could clarify whether specific dietary components promote or limit *Blastocystis* persistence and whether these dietary influences interact with other environmental and microbial factors. Integrating these analyses within longitudinal cohort studies would allow for a more comprehensive understanding of how diet modulates *Blastocystis*-host-microbiota dynamics over time. Moreover, studies should not only focus on *Blastocystis* but also consider other common parasites and luminal protists that may co-exist in individuals. These co-occurring organisms could interact in agonistic or synergistic ways, influencing the observed microbiota changes. Investigating these interactions would provide more precise assessments of *Blastocystis* intensity and yield deeper insights into the causal mechanisms and dynamics underlying its presence, as well as its broader impact on the host’s microbiota structure and composition.

We aimed to explore the eukaryotic microbiota concerning *Blastocystis* presence (Fig 2C, 2D). *Blastocystis* colonization was associated with specific features of the eukaryotic microbiota. In the *Blastocystis*-positive group, we observed an increased abundance of *Entamoeba coli*, a commensal protozoan often considered benign [52]. This contrasts with individuals negative for *Blastocystis*, who were found to have a higher abundance of *Saccharomyces cerevisiae* and *Candida albicans* (Fig 2D). There are a limited number of studies that involve the analysis of eukaryotic microbiota; however, changes in fungal communities have been reported in conditions such as irritable bowel syndrome (IBS), characterized by an enrichment of *Saccharomycetes* and a decrease in alpha diversity, which is consistent with our findings [53]. Furthermore, while *Candida albicans* is a normal component of the gut microbiota in healthy individuals, it has been associated with mucosal diseases and can translocate into the bloodstream, potentially leading to invasive, life-threatening infections, particularly in immunocompromised patients [54,55]. Additionally, *C. albicans* has been observed to contribute to the development of colitis in mouse models and shows increased abundance in patients with Crohn’s disease, although the pathogenic mechanisms remain not fully understood [56]. These findings suggest a potentially beneficial and complex interaction between *Blastocystis* and other eukaryotes; however, the level and type of interaction among *Blastocystis*, other microbial eukaryotes (including fungi), and the host microbiome remain unclear. Future studies should focus on exploring the eukaryotic component of the gut microbiome as a whole, rather than targeting individual microbial species, to better elucidate the role of eukaryotic microbiota within the gut ecosystem.

Further analysis regarding *Blastocystis* intensity groups revealed that the intensity of *Blastocystis* may be related to the abundance of specific eukaryotic taxa (Fig 3B). Higher *Blastocystis* intensity was linked with genera such as *Issatchenkia*, *Pleurostomophora*, and *Citeromyces*. These genera, likely stemming from the consumption of fermented foods and vegetables, may indicate a response to or modulation by elevated *Blastocystis* levels [53,57,58]. These genera, while not extensively studied, could contribute to the gut’s eukaryotic diversity by promoting an anaerobic environment and potentially interacting with other microbial components to maintain balance. However, their impact on these interactions requires further analysis to determine whether these changes are beneficial or detrimental to the host. The observed differences in eukaryotic taxa associated with *Blastocystis* presence and intensity underscore the protist’s potential role in shaping the eukaryotic microbiota [57,58]. Metagenomic and metabolomic approaches, particularly in longitudinal prospective studies, are crucial for characterizing all microbiota components and predicting functional changes. These methods allow for a comprehensive understanding of the mechanisms and direction of changes mediated by *Blastocystis* presence and intensity. By integrating these approaches, it will be possible to establish causal links, identify metabolic pathways affected by *Blastocystis*, and elucidate how these shifts impact host health, potentially revealing key insights into the protist’s role in modulating the gut ecosystem.

The random forest classifier’s ability to predict *Blastocystis* colonization status based on microbiota composition highlights its potential as a promising approach [59]. The model demonstrates superior performance compared to random chance, validating its utility in distinguishing between *Blastocystis*-positive and -negative individuals. The classifier’s performance, while showing some misclassification in training—particularly among positive samples—suggests that the model effectively identifies key microbial indicators associated with *Blastocystis* colonization. The model identified several taxa as critical for classifying *Blastocystis* status (Fig 4). Notably, and in line with the results presented earlier, *Faecalibacterium prausnitzii* and *Bacteroides ovatus* were among the top taxa with high Mean Decrease in Gini values. *Faecalibacterium prausnitzii* is well known for its anti-inflammatory properties and its critical role in maintaining gut health, which may correspond with the observed microbiota stability in the presence of *Blastocystis*. The prominence of these taxa suggests that *Blastocystis* colonization could be linked to the promotion of beneficial microbial communities that contribute to gut homeostasis [60–62]. Similarly, *Bacteroides ovatus* is involved in the breakdown of complex carbohydrates, contributing to a more balanced and diverse microbial environment. Likewise, *Bacteroides thetaiotaomicron* and *Ruminococcus bromii*, both significant in the classifier, are involved in complex carbohydrate digestion and the production of short-chain fatty acids, which are crucial for gut health and homeostasis [5,8,39,60–64]. *Entamoeba coli*, another important taxon discussed before, is a commensal eukaryote often found alongside *Blastocystis*. Its presence may indicate a balanced eukaryotic community that can coexist with *Blastocystis* without causing dysbiosis [3]. These findings suggest that the classifier could be a valuable tool for predicting *Blastocystis* colonization by analyzing the relative abundances of specific taxa. For instance, a high presence of *Faecalibacterium prausnitzii* or *Bacteroides ovatus* might indicate a higher likelihood of *Blastocystis* colonization. This predictive capability could provide key insights into microbial interactions in future research, potentially aiding in the development of targeted interventions or treatments aimed at maintaining or improving gut health. By leveraging these microbial signatures, we may better understand the functional dynamics of *Blastocystis* within the gut ecosystem.

It is important to acknowledge the limitations of our study. First, the sample size was relatively small, and we were unable to collect detailed information regarding key variables, such as diet. The hypothetical role of *Blastocystis* suggested through our analysis necessitates further investigation using longitudinal studies that incorporate metagenomic, metabolomic, and immunological approaches, alongside comprehensive data on dietary habits, lifestyle factors, and comorbidities. Such information is crucial for making accurate inferences about *Blastocystis*-host interactions, particularly considering the co-occurrence of other eukaryotes that may influence the observed changes [14,65]. Further research is needed to elucidate the mechanisms underlying the interactions between *Blastocystis* and the host microbiota. While our study employed a metabarcoding approach to characterize both bacterial and eukaryotic communities, this method does not enable predictions of metabolic and functional changes that may elucidate the dynamics involved in these interactions. It is important to highlight that metabarcoding analyses have limitations, such as the inability to determine organism viability (alive, dead, or dormant) or ecological role (transient vs. established). Additionally, primer bias and variations in 16S/18S gene copy numbers may affect relative abundance data, requiring cautious interpretation. Larger sample sizes and prospective longitudinal studies are essential to determine the directionality and causality of these relationships and validate *Blastocystis* intensity over time. Although we identified several relevant taxa, particularly within fungal communities, a deeper understanding of the significance of these specific taxa in the microbiota is necessary to clarify their potential positive impacts on host health. This comprehensive approach will enhance our ability to interpret the role of *Blastocystis* and its possible effects on the gut ecosystem. A potential limitation of our study is the exclusive focus on *Blastocystis* when evaluating associations with gut microbiota composition and diversity. Notably, *Blastocystis* was frequently linked to the presence of *Entamoeba coli* in our samples, suggesting that similar analyses should ideally be conducted for *E. coli*. If comparable results were observed for *E. coli*, it would highlight that *Blastocystis* is not the sole organism influencing the microbial patterns described in this study. Such findings could broaden our understanding of how co-occurring gut eukaryotes contribute to microbial ecosystem dynamics. Future studies incorporating detailed analyses of *E. coli* and other common eukaryotes are warranted to clarify the extent to which these interactions are organism-specific or reflect broader ecological phenomena. This also underscores the importance of considering multiple gut eukaryotes when interpreting microbiome studies, as their collective impact may provide a more comprehensive understanding of host-microbiota interactions. Subtyping of *Blastocystis* was not detailed, limiting our ability to assess potential subtype-specific effects on the microbiota. Additionally, although we focused on a healthy rural population, co-infections, and environmental factors, which may influence microbiome dynamics, were not systematically evaluated. Future studies incorporating high-resolution molecular techniques and broader epidemiological data will be essential to further elucidate these interactions.

Our findings have significant implications for understanding the role of *Blastocystis* in gut health. Rather than being a pathogen, *Blastocystis* may act as a commensal organism that supports a diverse and functionally rich gut microbiota [5–8]. The presence of *Blastocystis* was related to taxa that promote nutrient absorption, immune regulation, and protection against pathogens, all of which are critical for maintaining overall health. In non-westernized rural populations, where diet and environmental factors play a significant role in shaping the microbiota, *Blastocystis* may enhance the gut’s ability to adapt to these influences, contributing to a healthier microbiome [7]. *Blastocystis* may shape gut microbiota through nutrient competition, immune modulation, and metabolic shifts. It can alter microbial composition by competing for carbohydrates and nitrogen sources, influencing immune responses, and affecting metabolite production, such as short-chain fatty acids. These mechanisms could explain the microbiota differences observed in individuals with varying *Blastocystis* colonization intensities. Future transcriptomic and metabolomic studies will help clarify these interactions [7,8,40,42,45–47]. The potential for *Blastocystis* to modulate the microbiota beneficially also opens the door to new therapeutic approaches. By promoting the growth of beneficial bacteria and maintaining a balanced eukaryotic community, *Blastocystis* may serve as a marker of gut health and could be used as a target for microbiota-based therapies [7]. The findings of this study highlight the potential role of *Blastocystis* in shaping gut microbiota composition, which may have broader implications for global public health. Given its relationship with increased microbial diversity—often linked to gut resilience—future studies should explore whether *Blastocystis* can serve as a biomarker for microbiota stability in diverse populations. Additionally, its presence in healthy individuals raises the question of whether it could be leveraged in microbiota-based interventions, such as probiotic therapies or fecal microbiota transplantation strategies [7,8,40,42,45–47]. Integrating *Blastocystis* screening in epidemiological surveys and microbiome-based diagnostics could help refine our understanding of gut ecosystem dynamics, particularly in regions with high parasitic burden and nutritional challenges. Future research should focus on exploring the mechanisms by which *Blastocystis* interacts with the microbiota and the specific conditions under which it exerts its beneficial effects.

In conclusion, our study suggests that *Blastocystis* colonization is associated with a more diverse and functionally rich gut microbiota, challenging the traditional view of *Blastocystis* as a pathogen. The presence of *Blastocystis* was linked to taxa that play critical roles in gut health, particularly in non-westernized rural populations where diet and environmental exposures differ from urban settings. By fostering a diverse microbial community and promoting beneficial taxa, *Blastocystis* may act as a commensal organism that supports gut health and resilience. Given the observed relationships between *Blastocystis* colonization and gut microbiota diversity, future research should focus on experimental validation of its potential functional role. Longitudinal studies and controlled interventions could help determine whether *Blastocystis* actively contributes to gut microbial stability or merely reflects a healthy gut environment. Additionally, exploring its effects in germ-free or antibiotic-treated animal models could provide mechanistic insights. If *Blastocystis* proves to have beneficial interactions with the microbiota, it could be considered for microbiome-targeted therapies, particularly in conditions linked to dysbiosis, such as inflammatory bowel diseases or metabolic disorders. Further research is needed to fully understand the role of *Blastocystis* in gut microbiota dynamics and its potential implications for human health.

## Supporting information

S1 Fig Geographical sampling area corresponding to the rural region of Cauca, Colombia. The map was generated using QGIS 3.26.3 (Basemap: ESRI Terrain (XYZ Tiles); Sources: ESRI, DANE). The base layer of the map was obtained from the Departamento Administrativo Nacional de Estadística (DANE) Geoportal: https://geoportal.dane.gov.co/servicios/descarga-y-metadatos/datos-geoestadisticos/, specifically from the following dataset: https://geoportal.dane.gov.co/descargas/mgn_2022/MGN2022_DPTO_POLITICO.zip The dataset is publicly available in accordance with the policies and permissions of DANE, as stated: “In this section, the Departamento Administrativo Nacional de Estadística (DANE) makes the following information available to our information groups and the general public, in compliance with the Transparency Law and the Right of Access to National Public Information (Law 1712 of 2014) and MinTIC Resolution 1519 of 2020, which defines the standards and guidelines for publishing the information outlined in Law 1712 of 2014 and establishes requirements regarding access to public information, web accessibility, digital security, and open data.”
(TIFF)

S2 Fig Diversity metrics—observed richness, and Shannon indices—are compared between intensity groups of *Blastocystis*-positive samples, corresponding to (A) 16S rRNA data and (B) 18S rRNA data. Statistical analyses were performed using the Kruskal-Wallis (KW) test, followed by a post-hoc Mann-Whitney-Wilcoxon (MWW) test for multiple comparisons. The plots show interquartile ranges (IQR; boxes), medians (lines within the boxes), and the lowest and highest values within 1.5 times the IQR from the first and third quartiles (whiskers). This figure was created using RStudio software.
(TIFF)

S3 Fig Linear discriminant analysis effect size (LEfSe) identifying (A) bacterial and (B) eukaryotic taxa enriched in *Blastocystis*-positive and -negative groups, with a bar plot showing enriched taxa at the genus level. This figure was created using RStudio software.
(TIFF)

S1 Table Summary of sequence results and classification in *Blastocystis*-positive and -negative groups, as well as across different intensity groups. The table provides an overview of total reads, and classification accuracy for each group.
(XLSX)
