# Supplementary figures and images for: Impact of Blastocystis carriage and colonization intensity on gut microbiota composition in a non-westernized rural population from Colombia
Source: PLoS Negl Trop Dis. 2025 May 12;19(5):e0013111. doi: 10.1371/journal.pntd.0013111 (PMC12097710; doi:10.1371/journal.pntd.0013111)

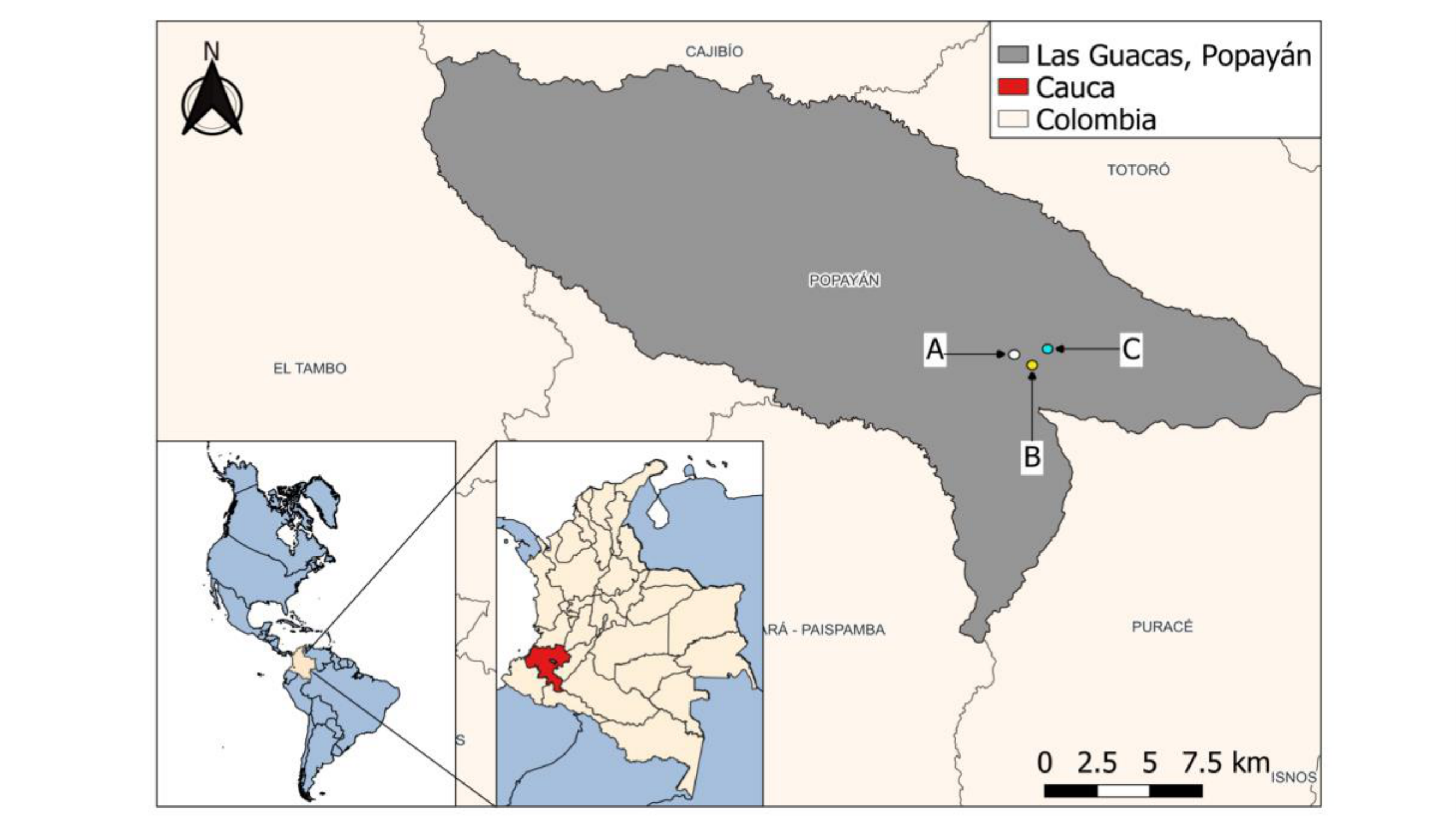

Supplement: S1 Fig — The map was generated using QGIS 3.26.3 (Basemap: ESRI Terrain (XYZ Tiles); Sources: ESRI, DANE). The base layer of the map was obtained from the Departamento Administrativo Nacional de Estadística (DANE) Geoportal: https://geoportal.dane.gov.co/servicios/descarga-y-metadatos/datos-geoestadisticos/, specifically from the following dataset: https://geoportal.dane.gov.co/descargas/mgn_2022/MGN2022_DPTO_POLITICO.zip The dataset is publicly available in accordance with the policies and permissions of DANE, as stated: “In this section, the Departamento Administrativo Nacional de Estadística (DANE) makes the following information available to our information groups and the general public, in compliance with the Transparency Law and the Right of Access to National Public Information (Law 1712 of 2014) and MinTIC Resolution 1519 of 2020, which defines the standards and guidelines for publishing the information outlined in Law 1712 of 2014 and establishes requirements regarding access to public information, web accessibility, digital security, and open data.” (TIFF) [file pntd.0013111.s001.tiff]

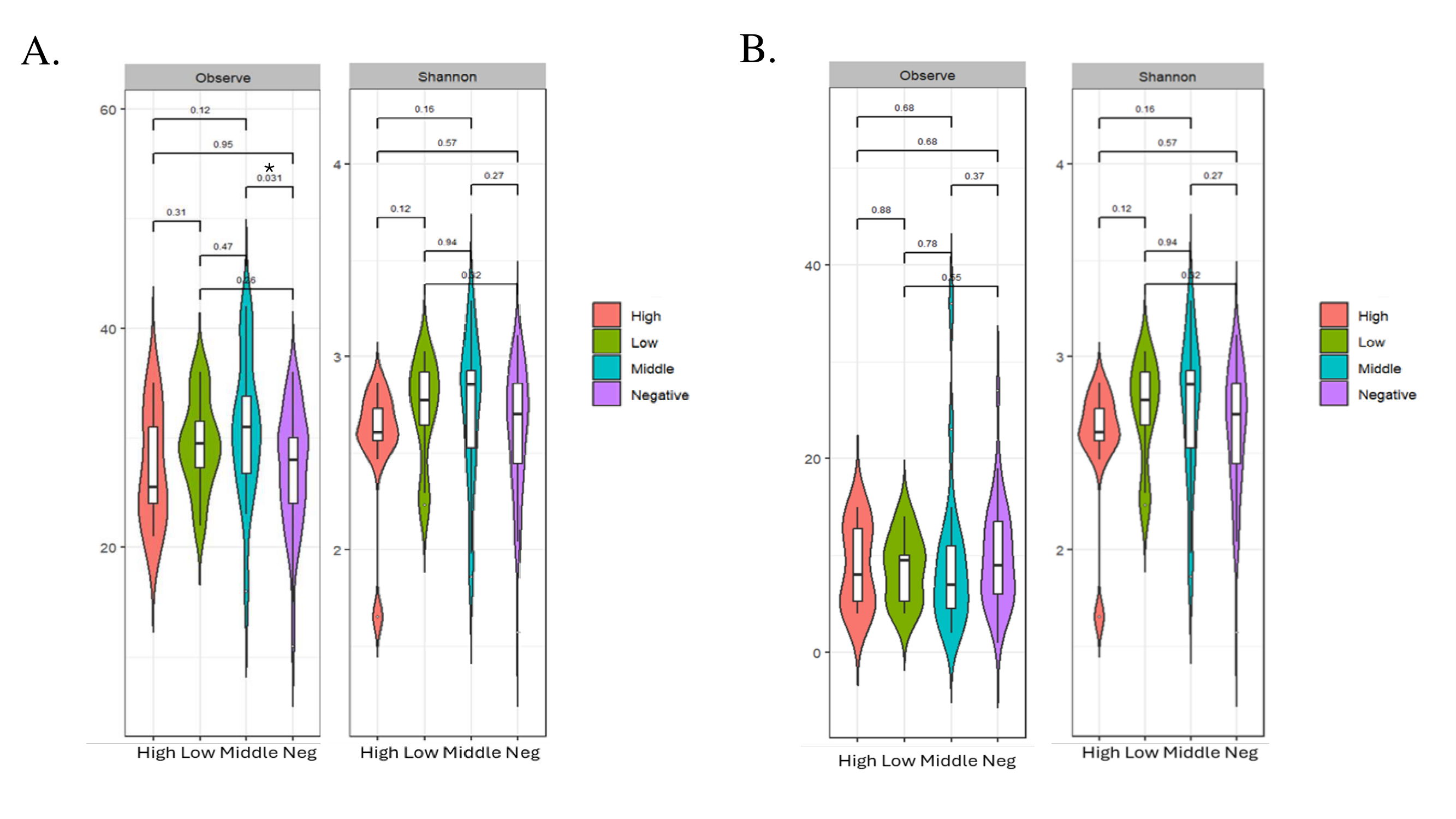

Supplement: S2 Fig — Statistical analyses were performed using the Kruskal-Wallis (KW) test, followed by a post-hoc Mann-Whitney-Wilcoxon (MWW) test for multiple comparisons. The plots show interquartile ranges (IQR; boxes), medians (lines within the boxes), and the lowest and highest values within 1.5 times the IQR from the first and third quartiles (whiskers). This figure was created using RStudio software. (TIFF) [file pntd.0013111.s002.tiff]

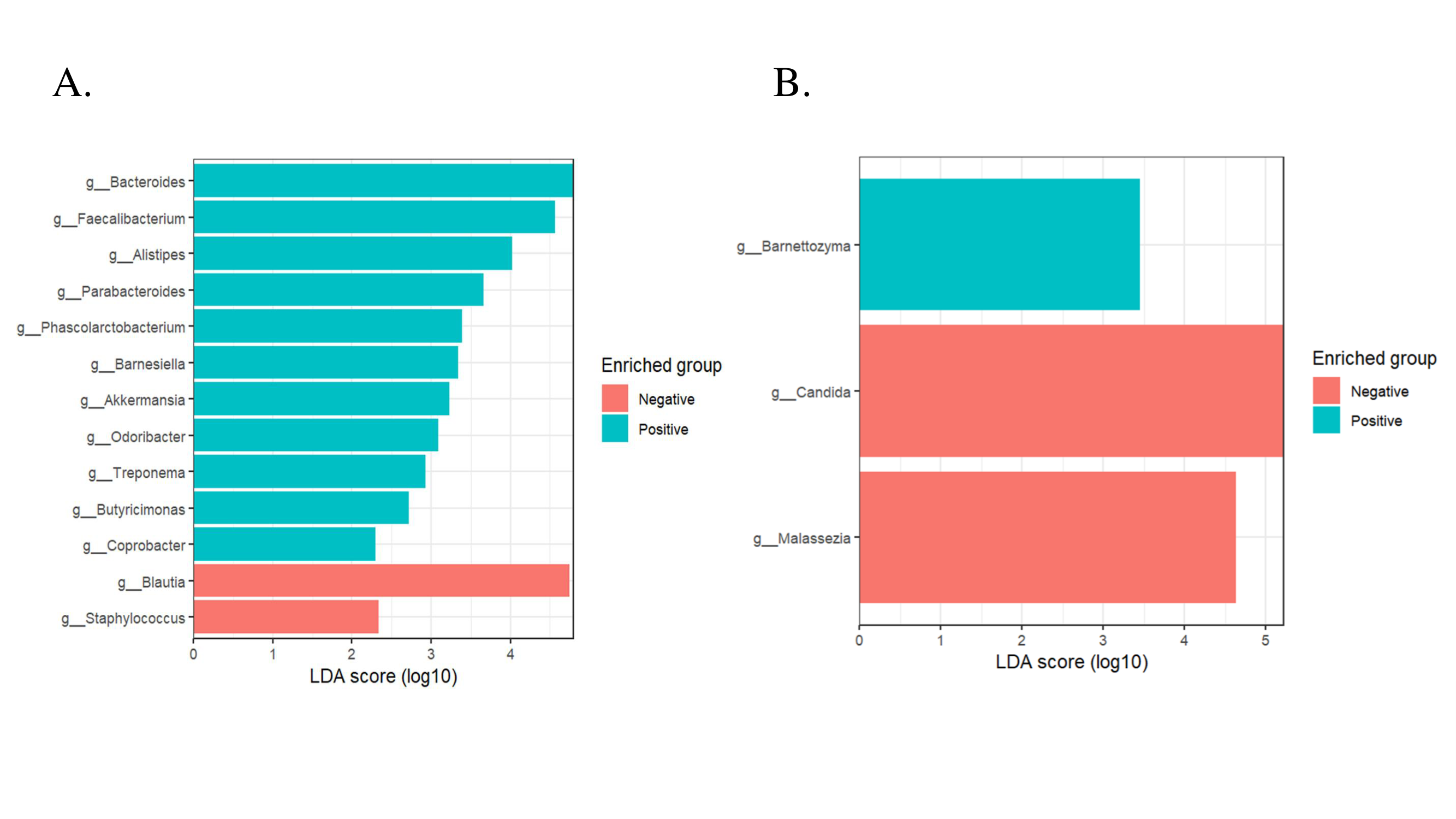

Supplement: S3 Fig — This figure was created using RStudio software. (TIFF) [file pntd.0013111.s003.tiff]
